# Community Drug Distributor Knowledge, Attitudes, and Motivation Surrounding Mass Drug Administration for Soil-Transmitted Helminths in India

**DOI:** 10.3389/fpubh.2021.714606

**Published:** 2021-11-23

**Authors:** Kumudha Aruldas, Saravanakumar Puthupalayam Kaliappan, Gideon John Israel, Gokila Palanisamy, Jabaselvi Johnson, Angelin Titus, Judd L. Walson, Arianna Rubin Means, Sitara Swarna Rao Ajjampur

**Affiliations:** ^1^The Wellcome Trust Research Laboratory, Division of Gastrointestinal Sciences, Christian Medical College, Vellore, India; ^2^Department of Global Health, University of Washington, Seattle, WA, United States; ^3^The DeWorm3 Project, University of Washington, Seattle, WA, United States

**Keywords:** drug distributors, mass drug administration (MDA), soil-transmitted helminth (STH), knowledge, attitude, motivation

## Abstract

**Background:** DeWorm3 is an ongoing multi-country community-based cluster-randomized trial assessing the feasibility of interrupting transmission of soil-transmitted helminths (STH) with community-wide mass drug administration (cMDA). In Tamil Nadu, India, community drug distributors (CDDs) worked with DeWorm3 field staff to counsel community members and deliver door-to-door deworming treatment. As CDDs were likely to influence successful delivery of cMDA, we describe drivers of CDDs' knowledge, attitudes, and motivation toward delivery of cMDA.

**Methods:** In this convergent mixed-methods study, a questionnaire on STH and cMDA was administered to 104 CDDs and 17 focus group discussions (FGDs) were conducted. Key outcomes in the quantitative and qualitative analyses included CDDs' knowledge about STH and cMDA and attitudes toward cMDA for STH. Univariate and multivariable logistic regression analyses were performed to determine the strength of associations between independent and outcome variables. The FGDs were analyzed using *a priori* thematic coding.

**Results:** CDDs who completed at least secondary school education [adjusted odds ratio (aOR): 2.71, 95% CI: 1.16–6.33] and had prior experience in health programs (aOR: 2.72, 95% CI: 1.15–6.44) were more knowledgeable about STH and cMDA. CDDs belonging to the scheduled castes and scheduled tribes (aOR: 2.37, 95% CI: 1.04–5.39), and to households engaged in a skilled occupation (aOR: 2.77, 95% CI: 1.21–6.34) had a more positive attitude toward cMDA for STH. The FGDs showed that while there were myths and misconceptions about STH, many CDDs believed that the adult population in their communities were infected with STH, and that a door-to-door drug delivery strategy would be optimal to reach adults.

**Conclusions:** Educational and socioeconomic backgrounds and experience in health programs should be considered while designing CDD trainings. Along with cMDA delivery for STH, as CDD do share community myths and misconceptions around STH, they should be proactively addressed during the CDD training to strengthen competency in counseling.

## Introduction

The current soil-transmitted helminths (STH) control strategy of the World Health Organization (WHO) targets deworming of pre-school (PSAC, 1–4 years), school-aged children (SAC, 5–14 years), and women of reproductive age including pregnant women in the second and third trimesters and breastfeeding women, and adults in certain high-risk occupations (1). While this strategy is unlikely to interrupt STH transmission, mathematical modeling and recent field trials indicate that it may be possible to interrupt STH transmission by expanding programs to include community wide deworming (2–5). To assess the feasibility of interrupting STH transmission, a large multi-country community-based cluster randomized trial, the DeWorm3 Project, is ongoing to compare biannual community-wide mass drug administration (cMDA) with albendazole to school-based targeted deworming. The study is implemented in three sites–Commune of Come, Benin, Timiri, and Jawadhu Hills in Tamil Nadu, India, and Mangochi District in Southern Region, Malawi.

In India, cMDA is delivered by community drug distributors (CDDs), who are either volunteers from the community, or equivalent community-based health workers called Accredited Social Health Activists (ASHAs) (6). ASHAs are women hired by the government to act as health educators and promoters in their communities. In order to implement cMDA with high coverage, a door-to-door treatment strategy by CDDs was adopted in the trial sites. Evidence suggests that CDDs are effective at delivering MDA with high coverage and compliance when they are members of the community that they are treating, female, and highly knowledgeable and effective communicators (7–10). CDDs engaged in lymphatic filariasis (LF), and onchocerciasis MDA programs in sub-Saharan Africa and South Asia, played a pivotal role in achieving the high treatment coverage required to reduce neglected tropical diseases (NTD)-related disabilities and interrupt disease transmission (11–13). Data from these successful programs suggests that when CDDs provided high quality information to community members, they were more likely to participate when they perceived that the CDDs were well-trained (14–16). Likewise, low CDD knowledge and poor communication lead to mistrust and lower compliance (17, 18). In India, evidence from maternal and child health programs suggests that mothers are more likely to adopt healthy behaviors (e.g., taking iron and folic acid tablets during pregnancy and implementing newborn care) when knowledgeable community health workers provided counseling (19–22). Thus, a strong knowledge base amongst community health workers and health volunteers is important not only for providing quality health education, but also for earning the trust of community members who engage with these frontline health workers.

Health worker motivation and attitudes toward implementation may also influence progress toward program goals. Health workers and CDDs who believed that MDA reduced LF in the community had a positive attitude toward implementing the LF-MDA programs (9, 23). Motivating factors for CDDs include gaining communication skills, community recognition and incentives, and perceived reduction in disease burden (9). In India, ASHA performance appears to be higher among ASHAs self-reporting feelings of social responsibility and self-efficacy (24).

Understanding the factors associated with CDDs' knowledge, attitudes, and motivation is necessary for high treatment coverage of community-based interventions such as cMDA. In this context, we aim to describe CDDs' knowledge, attitudes, and motivation for delivering cMDA in the DeWorm3 project and to identify factors associated with high levels of knowledge and motivation and more positive attitudes toward cMDA.

## Materials and Methods

### Study Setting

The DeWorm3 trial is a hybrid type I randomized trial with an implementation research component that aims to identify facilitating factors and the barriers to implementation of cMDA for future scaling-up strategies. Details of the trial have been published previously (6, 25, 26). In India, the DeWorm3 study is implemented in two blocks in the southern state of Tamil Nadu–the Timiri block in Vellore district and Jawadhu Hills block in Tiruvannamalai district. Timiri is a rural block with a population of 105,691, 55 village panchayats, four primary health centers, and 25 health sub-centers (27). Jawadhu Hills is a tribal block with a population of 51,999 and 11 village panchayats, two primary health centers, and 13 health sub-centers (28). Annual enumeration of the study site carried out between October and December 2019 showed a study population of 147,570 people residing in 38,115 households. Further sociodemographic details on the study site and population in India have also been published (27, 29).

### Community-Wide Mass Drug Administration

In India, the DeWorm3 study engaged 109 CDDs, with each CDD expected to reach and treat 20 households per day over 5–6 days at each treatment round. A 2-day training program was conducted before the first round of cMDA in February 2018 followed by a 1-day refresher training before each subsequent round for a total of six trainings. The CDDs received a kit that contained albendazole tablets by GlaxoSmithKline Pharmaceuticals Limited, 400 mg for age 2 years and above, and, sachets of powdered albendazole (200 mg) for children aged 1–2 years, a handout of inclusion and exclusion criteria for cMDA, a picture card about the process to be followed while dispensing the drug (Supplementary File 1), and a spoon for administering the drugs. CDDs were accompanied door-to-door by a trial field worker who documented the drug distribution in real-time using a mobile phone-based data collection system. The CDDs received 10 Indian Rupees (INR) per household as an incentive to dispense cMDA to all household members, leading to an earning of 200–250 INR (~$3 USD) per day.

### Study Design

**Figure 1:**
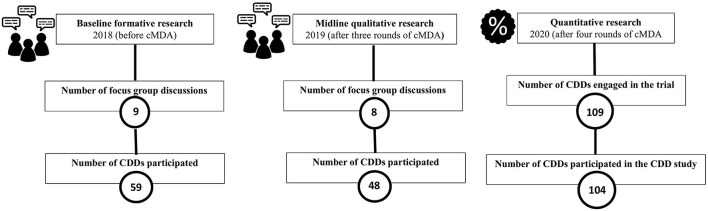
DeWorm3 CDD study design.

We conducted a convergent parallel mixed-methods (quant + QUAL) design. In the baseline formative qualitative data collected in February 2018 from nine focus group discussions (FGDs), 59 CDDs participated. In the midline qualitative research collected after three rounds of cMDA in March 2019 from eight FGDs, 48 CDDs participated (Figure 1). A questionnaire was administered to the CDDs after four rounds of training for cMDA. With 109 CDDs engaged in the trial, we had 78% power to detect a difference of 25% in CDDs' knowledge. Available literature on CDD engagement and STH knowledge was used to develop a 26-item questionnaire with a mixture of Likert scale, binary, and open-ended response options (30, 31). Questions to assess knowledge of STH (mode of transmission, signs, and symptoms of STH infections), knowledge about treatment (age-specific dosage and exclusion criteria), and CDDs' attitude (toward door-to-door directly observed treatment, perceived effectiveness of cMDA, and STH elimination) were included. CDDs' motivation was assessed with questions on the extent to which they liked working as CDDs and why, and their perceived level of confidence and satisfaction (Supplementary File 2). Four trained research assistants administered the paper-based questionnaire translated into the local language (Tamil) in March 2020 at the field offices. Evidence from nine FGDs conducted at the trial baseline (February 2018) and eight FGDs conducted at the midline (March 2019) are included. Qualitative questions focused on the training experience, knowledge and attitudes toward cMDA for STH, and their motivating factors to work as CDDs.

### Data Management and Statistical Analysis

Questionnaire data were entered using Epi Info™ 7.2 (Centers for Disease Control and Prevention, Atlanta, GA, USA) and data analysis was carried out with STATA 15.0 software (StataCorp, TX, USA). Analysis was grouped into three composite outcome variables, (i) sum of knowledge about STH and cMDA, (ii) sum of attitudes toward cMDA for STH, and (iii) sum of motivation to work as CDD in DeWorm3 cMDA. Considering all responses to be of equal weight, a score of 1 was given to each correct answer to calculate the total knowledge and attitude scores. The composite outcome variables were then transformed to binary outcome variables. For each outcome variable, individuals whose score was equal to or above the median were categorized as “high knowledge” or “positive attitude,” respectively, and those with scores below the median were categorized as “low knowledge” or “negative attitude.” Motivation variable was not included in the logistic regression analysis as there was no variation in responses to the motivation questions. Univariate logistic regression was performed to identify potential drivers of knowledge and attitude and independent variables that were significant (*P* < 0.05) were included in a multivariable logistic regression analysis that used a backward elimination procedure to build the final models. The independent variables used in the regression analyses were (i) socio-demographic characteristics such as age, gender, caste, education, and occupation, (ii) prior experience in health-related activities, (iii) membership of any community-based organizations, (iv) prior experience in health care delivery, and (v) prior participation in DeWorm3 activities. Chi-square test and Fisher's exact test (when the expected cell counts were <5) were also performed to compare ASHA and community volunteers. All FGDs were recorded on digital voice recorders, transcribed, and translated into English. The qualitative data were coded using *a priori* thematic coding and analyzed in ATLAS.ti 8.0.

## Results

### Background Characteristics of the CDDs

Of the 109 CDDs engaged in the trial in India, 104 CDDs (93 women and 11 men) participated in the study (Table 1). Their ages ranged from 19 to 62 years (median, IQR: 33, 28.0–37.5). Most were married (89%), Hindu (97%), and had lived in the area for ten or more years (89%). More than half of them belonged to disadvantaged socio-economic groups (listed in a schedule of the Indian constitution as scheduled caste or scheduled tribe, SC/ST) (58%) and were educated to the level of secondary school or more (64%). Nearly two-thirds of the CDDs (60%) were members of either a women's self-help group, a farmers' club, or a cooperative society. Most of them had attended two or more of the DeWorm3 CDD trainings (87%), participated in community sensitization activities held prior to cMDA to inform community members of upcoming treatment rounds (85%), and were involved in drug delivery during two or more rounds of DeWorm3 cMDA (89%).

**Table 1:** Characteristics of community drug distributors and comparison between ASHA and community volunteers.

**Variables**	***n* = 104**	**%**	**ASHA**	**Volunteers**	**Chi-square**
			***n* (%)**	***n* (%)**	***P-*value**
**Block**					
Timiri	69	66.4	13 (29.5)	56 (93.3)	–
Jawadhu Hills	35	33.6	31 (70.5)	4 (6.7)	
**Age**					
≥33 years	55	52.9	18 (40.9)	37 (61.7)	0.036
<33 years	49	47.1	26 (59.1)	23 (38.3)	
**Sex**					
Male	11	10.6	0 (0.0)	11 (18.3)	0.002^*^
Female	93	89.4	44 (100.0)	49 (81.7)	
**Marital status**					
Currently married	93	89.4	41 (93.2)	52 (86.7)	0.348^*^
Others (Unmarried/Widowed/Separated)	11	10.6	3 (6.8)	8 (13.3)	
**Education**					
Below secondary	37	35.6	18 (40.9)	19 (31.7)	0.331
Secondary and above	67	64.4	26 (59.1)	41 (68.3)	
**Religion**					
Hindu	101	97.1	44 (100.0)	57 (95.0)	0.261^*^
Others (Muslims/Christians)	3	2.9	0 (0.0)	3 (5.0)	
**Caste^#^**					
Others (MBC/BC/FC)	44	42.3	7 (15.9)	37 (61.7)	<0.01
SC/ST	60	57.7	37 (84.1)	23 (38.3)	
**Residence years in the village worked**					
<10 years	11	10.6	6 (13.6)	5 (8.3)	0.521^*^
≥10 years	93	89.4	38 (86.4)	55 (91.7)	
**Residence**					
Main village	68	65.4	29 (65.9)	39 (65.0)	0.923
Colony/Hamlet	36	34.6	15 (34.1)	21 (35.0)	
**CDD's main occupation**					
Unskilled/Semi-skilled work	43	41.4	0 (0.0)	43 (71.7)	<0.01
Skilled work	61	58.7	44 (100.0)	17 (28.3)	
**Highest level of household occupation**					
Unskilled/Semiskilled work	42	40.4	13 (29.6)	29 (48.3)	0.054
Skilled work	62	59.6	31 (70.5)	31 (51.7)	
**Worked on health, other than DeWorm3**					
No	35	33.7	0 (0.0)	35 (58.3)	<0.01
Yes	69	66.4	44 (100.0)	25 (41.7)	
**Years worked on health, other than DeWorm3**					
0–5 years	62	59.6	11 (25.0)	51 (85.0)	<0.01
>5 years	42	40.4	33 (75.0)	9 (15.0)	
**Member of any social group or union**					
No	42	40.4	22 (50.0)	20 (33.3)	0.087
Yes	62	59.6	22 (50.0)	40 (66.7)	
**Attended DeWorm3 CDD training**					
<2 trainings	14	13.5	2 (4.6)	12 (20.0)	0.023
≥2 trainings	90	86.5	42 (95.5)	48 (80.0)	
**Participated in DeWorm3 cMDAs**					
<2 cMDA	11	10.6	0 (0.0)	11 (18.3)	0.002^*^
≥2 cMDA	93	89.4	44 (100.0)	49 (81.7)	
**Place of drug distribution**					
Own village and other villages	86	82.7	34 (77.3)	52 (86.7)	0.211
Other villages only	18	17.3	10 (22.7)	8 (13.3)	
**Participated in DeWorm3 pre-cMDA community sensitization activities**					
Yes	88	84.6	40 (90.9)	48 (80.0)	0.128
No	16	15.4	4 (9.1)	12 (20.0)	

*cMDA, Community wide Mass Drug Administration*.

# *As per National Family Health Survey classification (http://rchiips.org/nfhs/factsheet_NFHS-5.shtml). MBC/BC/FC, Most Backward Class/Backward Class/Forward Class; SC/ST, Schedule Caste/Schedule Tribe; ASHA, Accredited Social Health Activist; CDD, Community drug distributor*.

* *Fisher's Exact*.

**Figure 2:**
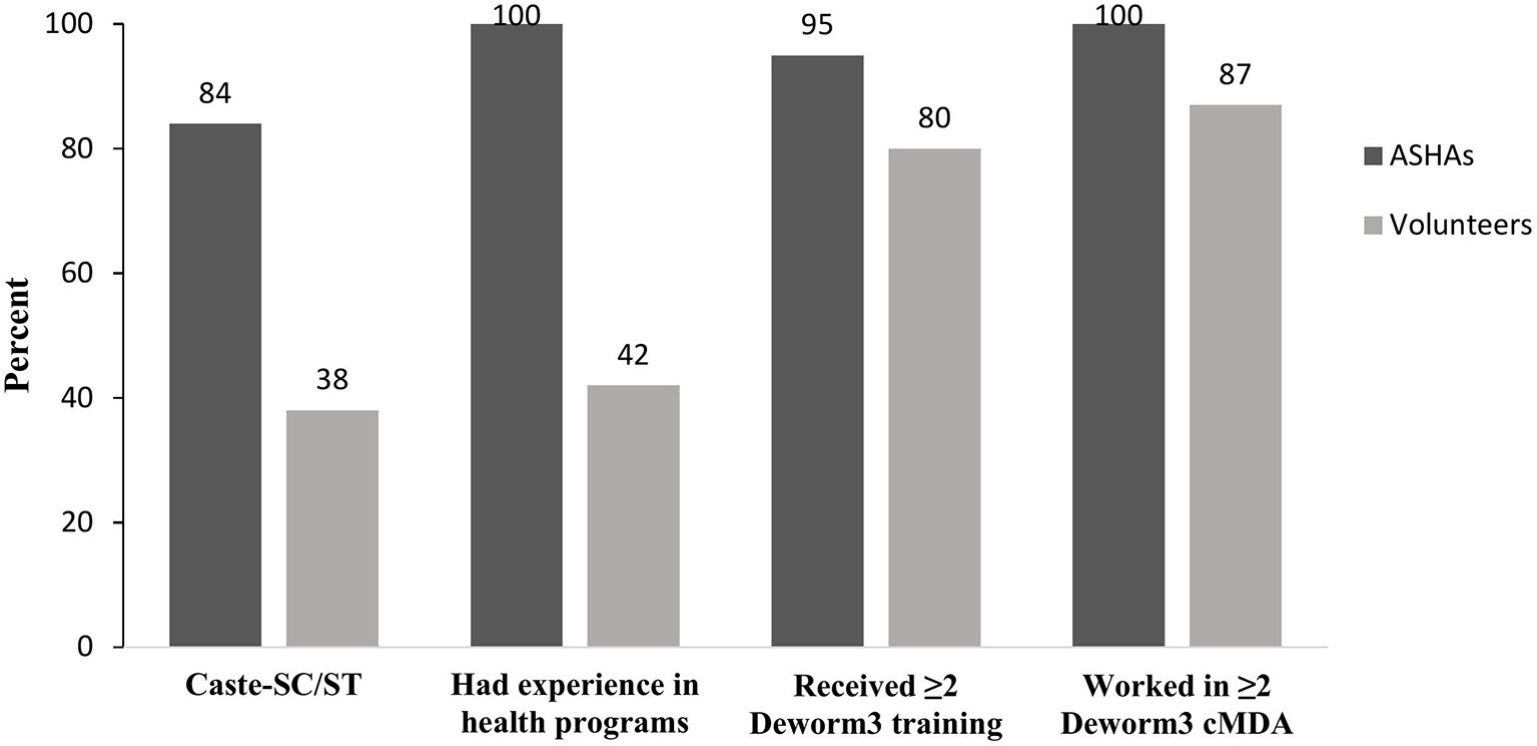
Differences in background characteristics between ASHAs and Volunteers.

Of the 104 CDDs, 42% had previously worked as ASHAs for government initiatives, including school National Deworming Day (NDD) (82%), pulse polio (100%), LF-MDA (73%), tuberculosis control program (48%), and maternal health (98%), child health (100%), and adolescent health (86%) programs. Most of these CDDs who were also ASHAs (31 of 44) lived in the Jawadhu Hills area. Among the 60 community volunteers (not ASHA) engaged as CDDs, less than half (42%) reported working in health programs including pulse polio (80%), LF-MDA (36%), child health (12%), dengue prevention and control program (12%), and NDD (4%). Several differences in the background characteristics between the ASHAs and the community volunteers were observed (Table 1). Significant difference between ASHAs and volunteers in their background characteristics is shown in Figure 2. A significantly higher proportion of ASHAs belonged to the SC/ST caste as they lived in the lower income Jawadhu Hills area (84% ASHA and 38% volunteers) (*P* < 0.01). A higher proportion of ASHAs had experience working in health programs than the volunteers (100% ASHA and 42% volunteers) (*P* < 0.01). More ASHAs had participated in two or more DeWorm3 trainings (96% ASHA and 80% volunteers) (*P* = 0.023) and two or more DeWorm3 cMDA rounds (100% ASHA and 82% volunteers) (*P* = 0.002) than volunteers.

### Knowledge of STH Infections and cMDA Among the CDDs

#### Knowledge of STH and Its Transmission

The formative qualitative research carried out with CDDs in February 2018 demonstrated that STH infections were commonly referred to as intestinal worms in the community and sometimes even as earthworms. CDDs across all clusters associated STH infection with open defecation and walking barefoot when the worms can get in through cracked heels. However, a few of the CDDs believed that intestinal worms are formed by eating sweets, meat, and eating raw rice.

“*Some children when they eat too much sweet, they get worms, if sweets are not taken, the worm will be somewhat less.”* (CDD # 2, Cluster 12)

“*If we eat raw rice, it is coming. They like eating raw rice*.” (CDD # 1, Cluster 15)

When the questionnaire was administered in March 2020 (after four CDD trainings had been completed), similar findings were observed. Only about half of the CDDs (53%) could name at least one type of STH as either hookworm, roundworm, or whipworm. Many referred to them as just intestinal worms (57%) and similar to the formative qualitative research, some even identified them as earthworms (22%). They correctly identified defecating in the open (77%), walking without footwear (77%), and not washing hands before eating (63%) as the three main modes of STH transmission.

#### Knowledge of Signs and Symptoms of STH Infections

In the qualitative research, CDDs from all the clusters correctly identified common signs and symptoms of STH including stomach pain, diarrhea, loss of appetite, and fatigue. They strongly associated STH infections with anemia as a serious consequence.

“*…. if the intestinal worms become more in the stomach, appetite will come down. If the appetite is less, then the blood level in the body will reduce*.” (CDD # 8, Cluster 15)

One of CDDs further elaborated that STH infections affect uterine growth leading to miscarriage and also the worms can eat up the embryo leading to infertility. Based on the questionnaire, CDDs identified appropriate signs and symptoms of STH including abdominal pain (61%), loss of appetite (55%), poor concentration (59%), anemia (31%), and diarrhea (23%).

#### Knowledge of Drug Dosage and Inclusion/Exclusion Criteria of cMDA

In FGDs, most CDDs had the knowledge of inclusion and exclusion criteria for treatment in cMDA. Some of them knew that children above the age of 1 year and pregnant women above 3 months of pregnancy can eat the tablet while a number of them excluded pregnant women. They said,

“*They said not to give for pregnant women; for those who have drunk liquor…. We should not give to very old people, those who are in bedridden and sick*.” (CDD # 5, Cluster 2)

The questionnaire administered confirmed that all of the CDDs knew the correct treatment dose for an adult as one tablet, and nearly all of them (95%) knew the treatment dose for children aged 1–2 years as half a tablet. Many of the CDDs were aware of the treatment criteria for cMDA; most knew that this included children above 1 year of age (75%), adults (80%), and pregnant women after their first trimester of pregnancy (69%). Most also knew who should not be treated, with the majority reporting that the sick or ill (87%) and intoxicated (55%) were ineligible for treatment. However, only about two-thirds of the CDDs (63%)–more ASHAs (84%) than volunteers (47%)–knew the name of the drug, albendazole, used in cMDA for STH. In the multivariable logistic regression analysis, completion of secondary school education (adjusted OR: 2.71, 95% CI: 1.16–6.33) and prior experience in health programs (adjusted OR 2.72, 95% CI: 1.15–6.44) was associated with a higher knowledge of STH and cMDA (Table 2).

**Table 2:** Multivariable analysis of factors associated with knowledge and attitude about STH and cMDA among community drug distributors.

**Variables**	***n* = 104**	**%**	**Knowledge about STH and cMDA**				**Attitude toward cMDA for STH**			
			**Univariable logistic**		**Multivariable logistic**		**Univariable logistic**		**Multivariable logistic**	
			**OR (95% CI)**	***P-*value**	**OR (95% CI)**	***P-*value**	**OR (95% CI)**	***P-*value**	**OR (95% CI)**	***P-*value**
**Age**										
≥33 years	55	52.9	Ref		–	–	Ref		–	–
<33 years	49	47.1	1.11 (0.51–2.41)	0.79	–	–	2.26 (1.02–4.99)	0.044	–	–
Sex^*^										
Male	11	10.6	–	–	–	–	–	–	–	–
Female	93	89.4	–	–	–	–	–	–	–	–
Marital status^*^										
Currently married	93	89.4	–	–	–	–	–	–	–	–
Others (Unmarried/Widowed/Separated)	11	10.6	–	–	–	–	–	–	–	–
**Education**										
Below secondary	37	35.6	Ref		Ref		Ref		–	–
Secondary and above	67	64.4	2.63 (1.15–5.99)	0.022	2.71 (1.16–6.33)	0.022	1.05 (0.47–2.35)	0.909	–	–
Religion^*^										
Hindu	101	97.1	–	–	–	–	–	–	–	–
Others (Muslims/Christians)	3	2.9	–	–	–	–	–	–	–	–
**Caste^#^**										
Others (MBC/BC/FC)	44	42.3	Ref		–	–	Ref		Ref	
Scheduled caste/scheduled tribe	60	57.7	1.09 (0.50–2.38)	0.83	–	–	2.27 (1.03–5.03)	0.043	2.37 (1.04–5.39)	0.040
Residence years in the village worked^*^										
<10 years	11	10.6	–	–	–	–	–	–	–	–
≥10 years	93	89.4	–	–	–	–	–	–	–	–
**Residence**										
Main village	68	65.4	Ref		–	–	Ref		–	–
Colony/Hamlet	36	34.6	1.40 (0.61–3.18)	0.426	–	–	0.88 (0.39–1.98)	0.768	–	–
**CDDs main occupation**										
Unskilled/Semi–skilled work	43	41.4	Ref		–	–	Ref		–	–
Skilled work	61	58.7	0.85 (0.39–1.87)	0.683	–	–	1.5 (0.69–3.31)	0.305	–	–
**Highest level of household occupation**										
Unskilled/Semiskilled work	42	40.4	Ref		–	–	Ref		Ref	
Skilled work	62	59.6	1.48 (0.67–3.26)	0.33	–	–	2.67 (1.19–5.99)	0.017	2.77 (1.21–6.34)	0.016
**Working as an ASHA**										
No	60	57.7	Ref		–	–	Ref		–	–
Yes	44	42.3	1.26 (0.58–2.77)	0.559	–	–	2.21 (0.99–4.94)	0.053	–	–
**Worked on health, other than DeWorm3**										
No	35	33.7	Ref		Ref		Ref		–	–
Yes	69	66.4	2.64 (1.14–6.09)	0.023	2.72 (1.15–6.44)	0.023	0.73 (0.32–1.66)	0.449	–	–
**Years worked on health, other than DeWorm3**										
0–5 years	62	59.6	Ref		–	–	Ref		–	–
>5 years	42	40.4	1.10 (0.50–2.42)	0.816	–	–	1.63 (0.73–3.61)	0.233	–	–
**Member of any social group or union**										
No	42	40.4	Ref		–	–	Ref		–	–
Yes	62	59.6	0.91 (0.41–2.01)	0.816	–	–	1.38 (0.63–3.04)	0.418	–	–
Attended DeWorm3 CDD training^*^										
<2 trainings	14	13.5	–	–	–	–	–	–	–	–
≥2 trainings	90	86.5	–	–	–	–	–	–	–	–
No. of DeWorm3 cMDAs participated^*^										
<2 cMDA	11	10.6	–	–	–	–	–	–	–	–
≥2 cMDA	93	89.4	–	–	–	–	–	–	–	–
Place of drug distribution^*^										
Own village/both villages	86	82.7	–	–	–	–	–	–	–	–
Other villages only	18	17.3	–	–	–	–	–	–	–	–
Participated in DeWorm3 pre-cMDA community sensitization activities^*^										
Yes	88	84.6	–	–	–	–	–	–	–	–
No	16	15.4	–	–	–	–	–	–	–	–

*cMDA, Community wide Mass Drug Administration; STH, Soil-transmitted helminthes; OR, Odds ratio; CI, Confidence interval*.

# *As per National Family Health Survey classification (http://rchiips.org/nfhs/factsheet_NFHS-5.shtml). MBC/BC/FC, Most Backward Class/Backward Class/Forward Class; ASHA, Accredited Social Health Activist; CDD, Community drug distributor*.

* *Logistic regression not performed if cell values were <10*.

### Attitudes of CDDs Toward cMDA

#### Beliefs About the Burden of STH Infections in the Community

CDDs' feedback in FGDs indicated that they believe STH infections might be widespread amongst adults in their communities. They perceived that adults were the source of STH infection and children were getting reinfected even though they were dewormed in school. They said,

“*It was spreading through children, so they gave (tablet) through school, now, it is spreading through adults. Because they are giving to adults, it will not spread through adults*.” (CDD # 1, Cluster 15)

“*It (worm) is there for adults as well as children. If children eat the tablet only the worm inside their stomach will be destroyed but it will not be destroyed for adults. So, if we give to adults, it is good for their whole family, right?*” (CDD # 4, Cluster 34)

The survey showed 72% of the CDDs believed that adults have STH infections and 56% estimating that at least half of the adult population in their communities were STH infected. The majority of CDDs (87%) accurately reported that a person could have an STH infection even if they do not feel like they do or if the worm is not visible to them. Over half of CDDs (55%) believed that STH infection is a serious disease.

#### Attitude About the Treatment Drug Used in cMDA for STH

Based on their field experience of distributing albendazole for STH, CDDs revealed that community members appreciated the effect of the drug although some of them had complained of side effect as diarrhea. Therefore, they believed that the treatment drug was effective. They said,

“*When we go again to give, they say that the tablet that you gave was good, you please give, and they will take in front of us.”* (CDD # 2, Cluster 13)

“*Worm moves in the stomach for them. When they defecate worm moves, now it is free. They are asking if you have a tablet, please give…. it is good for us.”* (CDD # 1, Cluster 13)

Based on the questionnaire, CDDs reported that albendazole is an effective (100%) and safe drug (99%) with no side-effects (99%). Some of the CDDs (38%), including the ASHAs (23%), mentioned other natural remedies for treating STH such as neem leaf paste, bitter gourd juice, aloe vera with rice, castor oil, and gingelly oil. The 33 CDDs who mentioned natural remedies for treating STH infections believed that they were “very effective” or “somewhat effective” in treating STH. A small percentage of the CDDs (2%), all of whom were volunteers, reported that STH infection actually helps in digestion and can eliminate other “bad” worms in the stomach.

#### Attitude of CDDs About the DeWorm3 cMDA and Its Delivery Strategy

The qualitative research showed a positive attitude of CDDs across all clusters. They said cMDA was advantageous because STH infection could be prevented when everyone eats the tablet at the same time, and people would generally feel healthy. Some said it would have been costly to buy deworming tablet but for DeWorm3 cMDA. They were in favor of implementing cMDA and door-to-door drug delivery strategy as otherwise not all would go to the centralized place to collect their tablets because they would be busy with their work. They said,

“*If we look at school, they (children) are regularly going to school. They would eat regularly once in 6 months. But those who are in the village will not get once in six months.”* (CDD # 2, Cluster 34) “*It will be better to give house to house because we can call all the people and meet directly. Otherwise, they will not come searching for us and take from us*.” (CDD # 5, Cluster 2)

The questionnaire confirmed that all the CDDs (100%) believed that it is “very necessary” or “necessary” to deliver cMDA with a door-to-door strategy.

#### Attitude of CDDs Toward Control and Elimination of STH by cMDA

Across many clusters, CDDs clearly believed that cMDA could prevent STH infection; otherwise, it would persist despite children being treated in schools. For example, they said,

“*If we give the tablet for school children and if the school children eat it, then we could interrupt the worm infection for children. If the members at home did not eat the tablet, and if the children at home defecate in the open place, in this situation, the worm will again spread.”* (CDD # 5, Cluster 34)

“*We have been deworming only the children, but children were getting it, again and again, therefore if we give to the adults also all will be dewormed, and this will be useful to the children and all*. (CDD # 5, Cluster 17)

“*Madam, if we give to the children, it will not be completely destroyed. So, by giving to adults also, the whole family gets benefit out of it*.” (CDD # 1, Cluster 13)

The questionnaire administered revealed that all CDDs felt that STH infections could be controlled (100%). Among the reasons for why STH could be controlled, CDDs mentioned people were consuming the tablet distributed during cMDA (81%), using toilets (48%), wearing footwear (44%), and practicing handwashing (38%). However, when asked about STH elimination, 22% reported that it was not achievable. The reasons they felt were that not all were using toilets (59%), practicing handwashing (27%), wearing footwear (23%), or eating the tablet distributed in the DeWorm3 cMDA (23%). They also indicated that despite treatment, many were still infected with worms (18%). The benefits of STH control they listed included: being free of infection and being healthy (86%), having an improved appetite (32%), being free from stomach pain/diarrhea (38%), and reducing anemia (35%). In the multivariable logistic regression analysis, belonging to a disadvantaged group (SC/ST) (aOR: 2.37, 95% CI: 1.04–5.39) and belonging to a household engaged in a skilled occupation (aOR: 2.77, 95% CI: 1.21–6.34) were significantly associated with CDDs having a positive attitude toward cMDA for STH.

### Motivation of CDDs

#### Confidence in Working as a CDD

The CDDs reported that DeWorm3 trainings helped them understand the process of delivering cMDA and build rapport with the community. They said,

“*One main thing is that, when we go to give the tablet, we should answer the people according to the questions they ask us, we should not get tensed. If we get tensed, people will get angry, and they will tell that they will not eat the tablet. We should give the tablet without any tension*.” (CDD # 4, Cluster 13)

The questionnaire confirmed the findings of the qualitative research, with CDDs reporting that they were “very confident” (92%) or “confident” (8%) working as CDDs and had all the resources and materials to function as a CDD (91%).

#### Satisfaction of and Interest in Working as a CDD

The midline FGDs revealed that the CDDs had become well-acquainted with the community members and felt a sense of carrying out community service by participating in cMDA. They said,

“*Only because we gave tablet, we go and talk to everyone…. They are called by pet names; we do not know the full name; when we give the tablet, we get to know their full name. We know the address, house number…”* (CDD # 4, Cluster 17)

“*When we gave the first time, some of them said - we don't want this tablet, go out, why are you coming. They chased us away, but when we give the second and the third time, they welcomed us. That gave us joy and strength.”* (CDD # 4, Cluster 17)

The questionnaire also confirmed this finding with CDDs being “very satisfied” (89%) or “satisfied” (11%) with their work, and they “very much liked” (93%) or “liked” (7%) working as a CDD. Reasons they selected included the (i) opportunity to serve the community (90%), (ii) feeling that the community recognized them (27%), (iii) monetary benefits (8%), and (iv) increase in their knowledge (7%). In midline qualitative research, the CDDs appreciated the monetary incentives they were getting, though, some of the CDDs expressed that the incentive was inadequate because they had to make multiple visits to some households for treatment delivery. They expressed as,

“*As of now, this itself is a great help for us, because of this we get some incentives on every month*.” (CDD # 3, Cluster 2)

“*But the incentive is not sufficient for us because we go to a house about 7 to 8 times.”* (CDD # 3, Cluster 2)

## Discussion

In this convergent mixed-methods study, we investigated the knowledge, attitudes, and motivation of CDDs engaged in carrying out cMDA during the DeWorm3 trial in southern India. Overall, they were knowledgeable with regards to STH and cMDA, had a positive attitude toward cMDA and were highly motivated to work as CDDs. Knowledge on STH transmission, treatment dosage, and the cMDA inclusion and exclusion criteria was high. The main factors associated with knowledge were education and prior experience in health programs. Knowledge of STH and cMDA was significantly higher among CDDs educated up to secondary school level or higher. Many of the CDDs were ASHAs, and an education level of secondary school is a criterion laid down by the Ministry of Health & Family Welfare for ASHA selection except in geographies with low levels of female literacy (32). Although association of CDDs knowledge with their level of education has not been reported in other studies on NTD, a LF program in Kenya considered high school completion as a criterion for selection as a drug distributor, and observed that MDA coverage was higher in areas where the CDDs had completed high school, implying that higher education of CDDs could be one of the factors driving higher compliance (33).

Evidence from this study suggests that there are remaining knowledge gaps and misconceptions, with some CDDs referring to STH as earthworms and others believing that eating sweets or raw rice causes infections. These beliefs may be the prevailing myths and misconceptions on STH where the CDDs reside. These misconceptions may reflect broader community myths, as shown in studies from China, Bangladesh, and Malaysia where community members believed that STH aid in digestion (34–36). Knowledge of treatment dosage of albendazole for STH was high but many did not know the name of the drug. However, more CDDs with prior experience of working in health programs knew the name of the drug. Many of the CDDs with prior experience of working in health programs had worked for the NDD and maternal and adolescent health programs that include anemia control measures which could potentially explain the higher level of knowledge of the treatment drug. This may have an implication for cMDA coverage, as an LF study from India has shown that community members did not trust taking LF drugs from volunteers who had poor knowledge of the drug administered (37). In Sri Lanka, the compliance in LF-MDA, defined as having ingested the drugs, was significantly higher (82%) among those who reported receiving information from CDDs about why they needed to take the treatment drug than those who were not informed (75%), indicating the crucial role of well-trained CDDs as counselors in MDA programs (14). In onchocerciasis MDA programs in Uganda and Ethiopia, compliance was likely to be 2.8–5.7 times higher when the community members perceived that the CDDs performed treatment related activities optimally and these included measuring height to determine the treatment dose, and delivering the treatment at their homes (15, 16).

The attitudes of CDDs toward cMDA for STH in our study was associated with their caste. CDDs belonging to the SC/ST caste had a significantly more positive attitude toward cMDA for STH, possibly because SC/ST households have a higher likelihood of exposure to STH infections due to poor availability of water and sanitation facilities and working in the fields leading to a higher risk perception (38). Programs targeting outreach based on the caste distribution of workers may improve coverage–the caste of health workers has been shown to limit the reach of LF programs and other health programs in India (37, 39). In contrast, a positive attitude toward cMDA among CDDs was also associated with skilled household occupation in our study. Many of the CDDs are ASHA workers from the tribal area and so categorized as SC/ST caste as well as belonging to households engaged in a skilled occupation and this could explain the overlap. CDDs reported that adult populations in their communities have STH infections, and some of them believed that STH might not be eliminated because not all in their communities were following safe sanitation practices. Studies conducted in the same areas where the CDDs resided showed low levels of improved sanitation facilities (34.6%) and 25.1% unweighted STH prevalence among adults (29). The FGDs with community women and men from the same communities also showed that adults had STH infections, and that embarrassment associated with having STH prevented them from seeking treatment (40). Income is shown to be a significant predictor of access to sanitation facilities, so CDDs belonging to households with a skilled occupation with higher income may have better access to sanitation and a better understanding of the association of STH with open defecation (41, 42).

In addition to the monetary benefit the CDDs received, other motivating factors to deliver cMDA for STH were mainly the opportunity to serve their community and recognition for their services by their community. This was especially true for community volunteers who are otherwise engaged in unskilled/semi-skilled work and therefore, probably not much recognized in their community. A systematic review of factors influencing motivation of CDDs in MDAs for control and elimination of NTDs demonstrated that community recognition and gain in knowledge were the main motivating factors (43). However, a high level of motivation and confidence of CDDs observed in our study may also be due to the continuous support they received from the DeWorm3 trial field workers. A prior systematic review of LF-MDA programs also indicated supportive supervision influenced the motivation and performance of drug distributors (13).

This study had several notable strengths, including the large size and the in-depth mixed methods utilized. However, there were also some limitations. We did not divide the FGD participants into ASHAs and volunteers, so we were unable to delineate the two groups views. Further, because many of the CDDs supported cMDA delivery in more than one village, it was not possible to correlate their knowledge, attitudes or motivation with treatment coverage achieved. As the survey questionnaire was administered to the CDDs by the research assistants, a potential response bias is also possible. Lastly, we did not conduct a beneficiary study to understand the community members perspectives of the CDDs capacity to inform and provide cMDA for STH.

## Conclusion

The results of this study suggest that, where feasible, engaging community health workers or volunteers with at least secondary level schooling and prior experience in health programs would be helpful for cMDA programs. As this may not be always feasible, CDD training programs should focus on developing competency-based skillsets specific to cMDA for STH. In addition, during training, the CDDs' knowledge and capacity for communication could be further strengthened by including a session addressing common myths and misconceptions prevailing in their communities around STH, disease burden, and severity of the disease. Training programs must ensure that all CDDs know the name of the treatment drug used, not just the dosage, particularly when tablets are directly delivered to beneficiaries without labeling. The cMDA programs could include activities to felicitate the CDDs during village meetings and events as community recognition is an important motivating factor for CDDs and will help build their standing as CDDs in the community that could then be leveraged across multiple programs. Selecting CDDs from the same community or with same caste background as program participants may facilitate achieving higher coverage as they would probably have better understanding of participant behaviors. Understanding CDDs' knowledge, attitudes, and motivation is important to design appropriate training materials and incorporate activities within the program to build their competency and motivation. Further, it would be helpful to measure if CDDs who have more knowledge or a more positive attitude indeed deliver cMDA at higher coverage.

## Data Availability Statement

The original contributions presented in the study are included in the article/Supplementary Material, further inquiries can be directed to the corresponding author.

## Ethics Statement

The studies involving human participants were reviewed and approved by the Christian Medical College, Vellore, India. The patients/participants provided their written informed consent to participate in this study. Written informed consent was obtained from the individual(s) for the publication of any potentially identifiable images or data included in this article.

## Author Contributions

KA, SPK, and SSRA conceived and designed the study. KA, SPK, and ARM designed the tools. GP, JJ, and AT collected the data. SPK, GJI, GP, KA, AT, and JJ analyzed the data. KA, SPK, SSRA, ARM, and JLW wrote and edited the paper. All authors reviewed and edited the manuscript.

## Funding

The DeWorm3 Project was funded by a grant from the Bill & Melinda Gates Foundation (OPP1129535, PI: JLW). The funders had no role in study design, data collection and analysis, decision to publish, or preparation of the manuscript.

## Conflict of Interest

The authors declare that the research was conducted in the absence of any commercial or financial relationships that could be construed as a potential conflict of interest.

## Publisher's Note

All claims expressed in this article are solely those of the authors and do not necessarily represent those of their affiliated organizations, or those of the publisher, the editors and the reviewers. Any product that may be evaluated in this article, or claim that may be made by its manufacturer, is not guaranteed or endorsed by the publisher.
