# Supplementary figures and images for: Community Drug Distributor Knowledge, Attitudes, and Motivation Surrounding Mass Drug Administration for Soil-Transmitted Helminths in India
Source: Front Public Health. 2021 Nov 23;9:714606. doi: 10.3389/fpubh.2021.714606 (PMC8650093; doi:10.3389/fpubh.2021.714606)

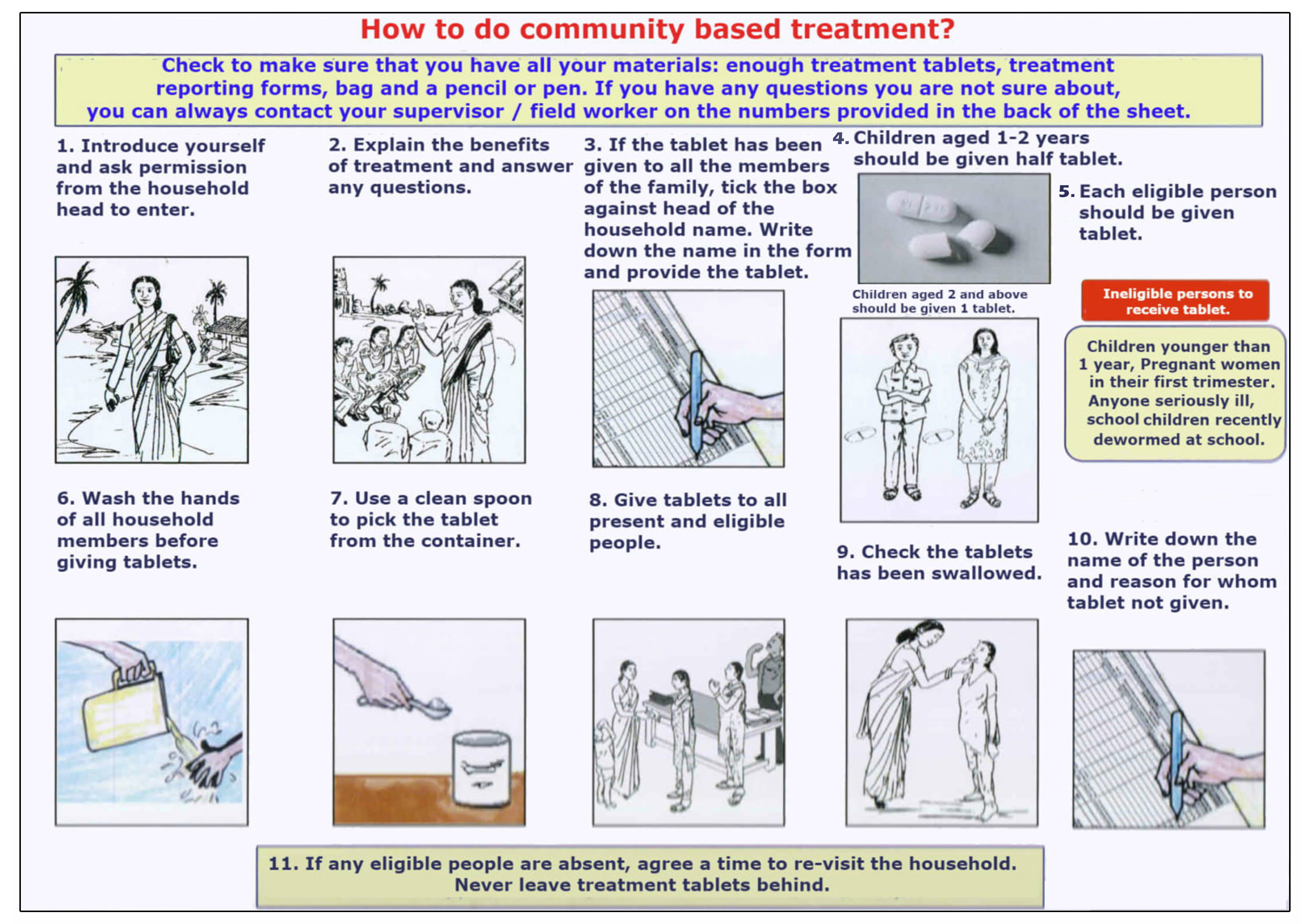

Supplement: Supplementary file 1 [file Image_1.jpg]
