# Supplementary material for: Community Drug Distributor Knowledge, Attitudes, and Motivation Surrounding Mass Drug Administration for Soil-Transmitted Helminths in India
Source: Front Public Health. 2021 Nov 23;9:714606. doi: 10.3389/fpubh.2021.714606 (PMC8650093; doi:10.3389/fpubh.2021.714606)
Supplement: Supplementary file 2 [file Table_2.pdf]

Supplementary file: 2

S. No	Questions
**Knowledge of STH and cMDA for STH**	
1.	What are the types of soil-transmitted helminths?
2.	How do these STH infections spread?
3.	What are the signs and symptoms of having intestinal worms?
4.	What is the name of the tablet distributed by DeWorm3 study in CWMDA for STH?
5.	Who all can take this tablet that is distributed by DeWorm3 study?
6.	How many tablets distributed in DeWorm3 study, should be given to a child 1-2 years of age?
7.	How many tablets distributed in DeWorm3 study, should be given to a 4 years old child?
8.	How many tablets distributed in DeWorm3 study, should be given to a person 25 years of age?
9.	Are there any advantages or positive effects of having STH infection?
**Attitude towards MDA for STH**	
10.	Do you think STH infection is a serious disease?
11.	Now you distributed this tablet given by DeWorm3 project house to house. In your opinion, how necessary it is that this tablet should be distributed only house to house?
12.	In a community-wide mass drug administration for STH program, in your opinion, when a person feels that they do not have intestinal worms then how necessary is it for a person to eat the deworming tablet?
13.	In your opinion, how important it is to make people eat the tablet only in front of you?
14.	Can a person have intestinal worms even if they do not feel it or it is not visible to them?
15.	STH are minor infections that cause no major harm to the body. To what extent do you agree or disagree with this statement?
16.	STH mainly affects children and not adults. To what extent do you agree or disagree with this statement?
17.	In your opinion, if a person always uses toilet, then how important it is for him/her to make take the tablet when it is distributed to everyone in the community?
18.	In your opinion, is the tablet for STH infections distributed in DeWorm3 study is an effective drug?
19.	In your opinion, is the tablet for STH infections distributed in DeWorm3 study is a safe drug?
20.	In your opinion, can STH infections be controlled?
21.	In your opinion, can STH infections be eliminated?
**Motivation and satisfaction of working as a CDD**	
22.	To what extent do you like working as a CDD in the DeWorm3 project?
23.	What do you like about working as a CDD in the DeWorm3 project?
24.	To work as a CDD in the DeWorm3 project for a community-wide mass drug administration for STH, how confident do you feel?

25.	To work as a CDD in the DeWorm3 project for a community-wide mass drug administration for STH, to what extent do you feel that you have the resources and materials?
26.	To what extent are you satisfied or not satisfied by working as a CDD of the DeWorm3 project?
